# Association between Dietary Inflammatory Index and Sarcopenia in Crohn’s Disease Patients

**DOI:** 10.3390/nu14040901

**Published:** 2022-02-21

**Authors:** Dongsheng Bian, Xutong Liu, Cenyu Wang, Yongmei Jiang, Yubei Gu, Jie Zhong, Yongmei Shi

**Affiliations:** 1 Department of Clinical Nutrition, Ruijin Hospital, Shanghai Jiao Tong University School of Medicine, Shanghai 200025, China; bds04159@rjh.com.cn (D.B.); jym10860@rjh.com.cn (Y.J.); 2 School of Public Health, Shanghai Jiao Tong University School of Medicine, Shanghai 200025, China; 3 Department of Nutrition, Shanghai Jiao Tong University School of Medicine, Shanghai 200025, China; liuxutong12@sina.com (X.L.); urpolaris@sjtu.edu.cn (C.W.); 4 Department of Gastroenterology, Ruijin Hospital, Shanghai Jiao Tong University School of Medicine, Shanghai 200025, China; gyb11809@rjh.com.cn (Y.G.); zj10455@rjh.com.cn (J.Z.)

**Keywords:** Crohn’s disease, dietary inflammatory index, sarcopenia, malnutrition

## Abstract

Background: Chronic inflammation is a pathophysiological cause of sarcopenia in Crohn’s disease (CD) patients. However, the potential impact of diet-related inflammation on sarcopenia has not yet been adequately investigated. We examined the associations between Dietary Inflammatory Index (DII) and sarcopenia in CD patients. Methods: A total of 140 CD patients from Ruijin Hospital in Shanghai were included in this cross-sectional study. DII scores were calculated from the dietary data collected using a validated food frequency questionnaire (FFQ). Sarcopenia was determined according to the Asian Working Group for Sarcopenia. Multivariable logistic regression analyses were performed to determine the association between DII and sarcopenia. Results: The mean DII score was 0.81 ± 2.13, ranging from −3.24 to 4.89. The overall prevalence of sarcopenia was 26.4%. The higher DII score significantly increased the risk of sarcopenia in CD patients (OR_Quartile4vs1_: 9.59, 95% CI: 1.69, 54.42, *p*_trend_ = 0.031) in the multivariable model after adjusting for more potential confounders. Moreover, CD patients with a lower DII had a significantly higher appendicular skeletal muscle mass index (ASMI, OR_Quartile4vs1_: 5.48, 95% CI: 1.51, 19.87, *p*_trend_ = 0.018) after adjusting for age, gender, BMI, smoking status and drinking status model. Yet, there were no significant differences between DII and ASMI after adjusting for more potential confounders. Additionally, no significant association was observed between DII and handgrip strength in the multivariable-adjusted models. Conclusions: Pro-inflammatory diet was associated with increased risk of sarcopenia in CD patients. CD patients should have a proper intake of energy and protein. These patients could also benefit from supplementation with enteral nutrition due to its anti-inflammatory potential.

## 1. Introduction

Inflammatory bowel disease (IBD), which refers to Crohn’s disease (CD) and ulcerative colitis (UC) jointly, is the representative chronic inflammatory disease of the gastrointestinal system [1]. The global emergence of CD and UC has highlighted the impact of changing environmental factors on disease development in a particular diet. Food components can affect the incidence of IBD and its development through modulation of intestinal microbiota, intestinal immune system, and inflammation [2].

As most IBD patients reported specific changes in their diet, it is believed that dietary factors might exacerbate symptoms as well as inflammation [3,4]. Some researchers showed that the inflammatory state could be modulated by food components affecting the progression and exacerbation of IBD via their effect on inflammation [5,6,7]. In addition, de Vries et al. showed that nearly 60% of IBD patients valued diet to be either more or equally important compared to their medicine, while more than 60% believed IBD decreases appetite [8]. Furthermore, our previous study revealed that the total energy and protein intake was inadequate among IBD patients in China. We also found a positive correlation between energy, protein, carbohydrate, and iron intake with fat-free mass [9]. When IBD patients suffer from chronic inadequate intake and symptoms such as diarrhea, it could increase the risk of malnutrition and the loss of skeletal muscle mass [10].

Sarcopenia, commonly found in the IBD population [11], is characterized by the degenerative loss of skeletal muscle mass, quality, and strength [12]. Patients with sarcopenia are more prone to falls and have a higher risk of death [13]. Furthermore, they may become more likely to suffer disability and hospitalization [14]. A study on physiological mechanisms indicated that inflammatory cytokines might activate the molecular pathway, causing disorder between protein synthesis and catabolism [15,16]. According to a meta-analysis on inflammation and sarcopenia, inflammatory indicator levels are negatively related to muscle strength and mass, while chronic inflammation has a critical role in sarcopenia. Consequently, assessing the correlation between inflammation, diet, and sarcopenia is a very important topic.

Existing data show that inflammatory markers, like interleukin-6 (IL-6), tumor necrosis factor-alpha (TNF-α), and C-reactive protein (CRP), have close contact with dietary intake [17,18]. Dietary Inflammatory Index (DII) is an appraisal tool that focuses on the inflammatory potential of the diet rather than on each specific nutrient. DII is a population-based priori index derived from a literature review of 1943 articles and is based on 45 dietary components of different weights, to sum up to the final score [19]. A higher DII score signifies a more pro-inflammatory diet, and a lower DII score signifies a more anti-inflammatory diet. Recent studies have reported a close association between DII scores and cardiovascular disease [20,21], frailty [22], metabolic syndrome [23], and fractures [24,25].

As inflammation may be the pathogenesis of sarcopenia, and DII can be reflected the inflammatory state in the body, it remains unclear if there is any relationship between DII and sarcopenia. However, studies on the association between DII scores and sarcopenia in IBD patients are limited. Consequently, this study aimed to investigate the association between sarcopenia and inflammatory potential of diets in CD patients by using the DII.

## 2. Materials and Methods

### 2.1. Participants

A total of 179 CD patients who received anti-TNF-α therapy at Shanghai Ruijin Hospital between July 2019 and June 2020 were enrolled. All CD patients were diagnosed with CD by their IBD specialists and according to the criteria established by the Inflammatory Bowel Disease Group of the Chinese Society of Gastroenterology, which included clinical characteristics, endoscopic, histological, and radiological examination et al.

**Figure 1:**
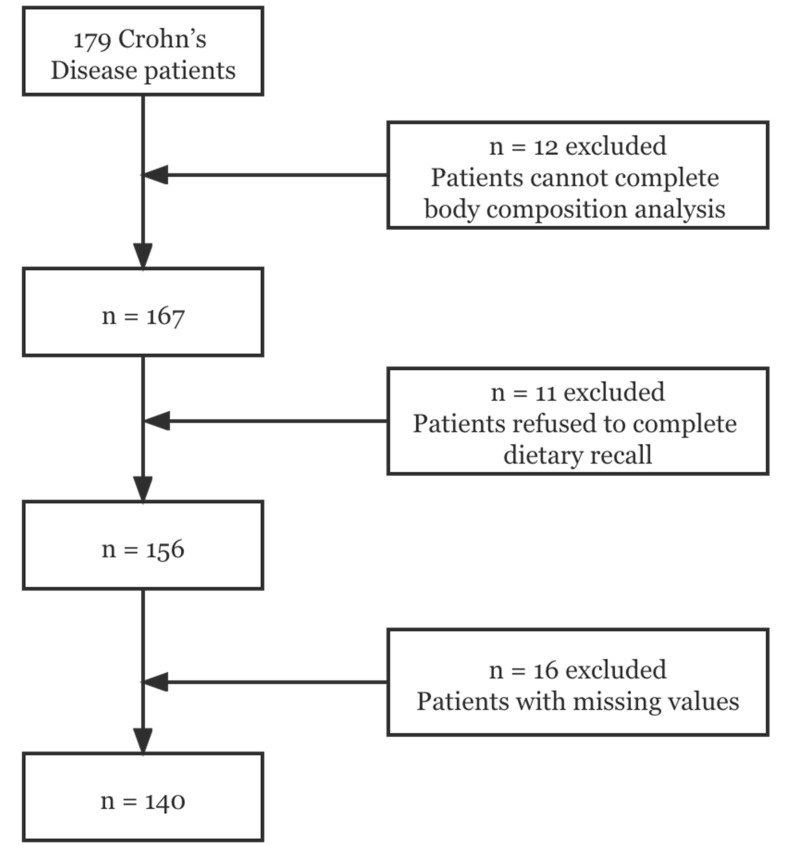
Flow chart of participants.

The exclusion criteria were the following: (I) patients aged < 18 years; (II) patients with a history of surgery within the last 3 months; (III) pregnant or lactating women; (IV) patients with chronic wasting diseases such as malignant tumors, poorly controlled diabetes, complex heart disease; (V) patients with metal implants (such as cardiac pacemakers) that could influence bioelectrical impedance results. In addition, those on special diets (e.g., Vegan, Atkins) were also excluded from this study. Thirty-nine patients were excluded due to missing values in the variables of body composition, food frequency questionnaire, or others, resulting in a final sample of 140 patients (Figure 1). All researchers involved in this study received professional training.

All participants signed informed consent. This study was approved by the Human Ethics Research Committee of Ruijin Hospital.

### 2.2. Data Collection

The following necessary information was collected through the interview: (I) sociodemographic data regarding age, gender, smoking, and drinking history; (II) disease characteristics, including disease duration and time of the first diagnosis. In addition, disease activity was assessed by Crohn’s disease activity index (CDAI). Patients with CDAI ≤ 150 were considered in clinical remission, whereas those with CDAI >150 were considered in the active phase; (III) The Montreal classification was used to define disease location as L1: terminal ileum, L2: colon, L3: ileocolon, and L4: involving the upper gastrointestinal tract. Disease behavior was defined as B1: non-stricturing and non-penetrating, B2: stricturing, and B3: penetrating; (IV) Anthropometric measurements were obtained using a digital scale to measure the participant’s weight to the nearest 0.1 kg (light clothes and shoes removed) and a stadiometer to measure the participant’s height to the nearest 1.0 cm (shoes removed). Body mass index was calculated as weight in kilograms divided by the square of height in meters (BMI, kg/m^2^), and it was categorized as underweight (BMI < 18.5 kg/m^2^), normal (18.5 ≤ BMI ≤ 24 kg/m^2^) and overweight (BMI > 24 kg/m^2^) according to the Chinese reference.

### 2.3. Dietary Intake Measurement

Diet was assessed using the Food Frequency Questionnaire (FFQ). Food frequency questionnaires have long been adopted as a standardized way to measure dietary intake. The questionnaires take a variety of forms. The questionnaire used in the present study included 27 groups of foods and beverages, which covered the majority of foods commonly consumed in China. Three aspects of each item were listed in the questionnaire, including whether the item was consumed, the usual frequency of consumption (number of times per day/week/month/year), and the estimated amount of food eaten each time, expressed using the local unit liang for weight (1 liang = 50 g) or cup for volume (1 cup = 250 mL).

Besides, nutrients and total energy intake were calculated by multiplying the usual frequency and portion size of each food item by the nutrient content using the food composition values from China Food Composition (National Institute of Nutrition and Food Safety, China CDC).

### 2.4. Dietary Inflammatory Index

DII, created by researchers at the University of South Carolina Cancer Prevention and Control Program [19], is an index used to predict the inflammatory potential of an individual’s diet. Based on six inflammatory biomarkers (IL-1β, IL-4, IL-6, IL-10, TNF-α, and CRP), DII was a priori derived from the literature and originally consisted of 45 food items and nutrients. In this study, we calculated DII based on 28 food parameters, including alcohol, vitamin B12, vitamin B6, β-carotene, carbohydrate, cholesterol, energy, total fat, fiber, folic acid, iron, magnesium, monounsaturated fatty acids, niacin, n-3 fatty acids, n-6 fatty acids, protein, polyunsaturated fatty acids, riboflavin, saturated fat, selenium, thiamin, vitamin A, vitamin C, vitamin D, vitamin E, zinc and isoflavones. To calculate the final score of DII, the individual’s daily intake of each food parameter was subtracted by the ‘standard global mean’ and then divided by this value’s standard deviation to obtain ‘z’ scores. These ‘z’ scores were then converted into percentile scores, doubled, and subtracted with 1 for the purpose of minimizing the effect of ‘right skewing’ [19]. By multiplying the inflammatory effect score, which was derived from the literature review, these processed values were summed up to obtain the overall DII score. A lower DII score indicated a more anti-inflammatory diet, while a higher DII score indicated a more pro-inflammatory diet.

### 2.5. Sarcopenia

We assessed body composition by multifrequency bioelectrical impedance analysis using the InBody S10 body water analyzer (Biopace Co., Ltd., Seoul, Korea), as this approach has been validated in China. This method automatically and simultaneously measures body weight, body mass index (BMI), and skeletal muscle mass.

A myometer was used to test handgrip strength, where measurement was performed with the participant in the following position: seated on an armless chair, with feet supported on the floor, hips and knees flexed at 90°, arms parallel to the body, elbows flexed at 90°, and forearms and wrists in a neutral position. The dominant side was measured three times at 1-min intervals, with verbal stimulation applied. The results were expressed in kilogram-force (kg), and the mean of the three measures was used.

The diagnosis of sarcopenia was established following the criteria stated in the Asian Working Group for Sarcopenia (AWGS) 2019 Consensus [26]. The appendicular skeletal muscle mass index (ASMI) was calculated as ASM divided by its corresponding squared height. Low muscle mass (ASMI< 7.0 kg/m^2^ for male, ASMI < 5.7 kg/m^2^ for female, via BIA) and low handgrip strength (handgrip strength< 28 kg for male, handgrip strength < 18 kg for female) were identified as sarcopenia.

### 2.6. Nutritional Assessment

The assessment was conducted using the Scored Patient-Generated Subjective Global Assessment (PG-SGA), which included weight history, food intake, symptoms, activities and function, disease and its relation to nutritional requirements, metabolic demand, and physical exam consisting of seven parts. The total score of 0–1 indicated no intervention required, a score of 2–3 indicated education or pharmacologic intervention required, a score of 4–8 indicated intervention by dietitian was required, a score of >9 indicated a critical need for nutrient intervention options. We classified patients with scores ≥4 as the malnutrition group and those with <4 as the normal group [27].

### 2.7. Statistical Analysis

SPSS22.0 was used for all the statistical analyses. Attributes data were expressed as a percentage of occurrence; variables data were expressed as mean and standard deviation (SD). Participants were classified into quartiles based on DII, Chi-square tests, and ANOVA were used to identify the significant differences in variables data across the quartiles. The relation between the DII and sarcopenia was tested in 3 models. Model 1 was an unadjusted regression model. Model 2 was adjusted for age, sex, BMI, smoking status, drinking status. Besides a potential role of malnutrition in sarcopenia of IBD patients, as well as disease activity, disease location, and behavior suggested by previous studies, we also tested whether the association between DII and sarcopenia in CD patients was independent of these factors by performing an additional model that further adjusted for these potential factors [28,29,30]. Model 3 was additionally adjusted for nutritional status, energy intake, disease duration, disease activity, Montreal classification-location and behavior based on Model 2. The trend of association was assessed using quartiles of DII as continuous variables.

## 3. Results

### 3.1. Characteristics of the Participants

Among the 140 CD participants, 72.14% were men, and the mean age was 32.59 ± 10.24 years. The mean DII score was 0.81 ± 2.13, ranging from −3.24 to 4.89. The mean PG-SGA score was 4.07 ± 3.68; there were 63 patients with PG-SGA score ≥ 4 who suffered malnutrition. The mean disease duration was 5.57 ± 5.29 years. Among males, the mean handgrip strength was 35.11 ± 1.53 kg, ASMI was 7.74 ± 0.17 kg/m2, while among females, the mean handgrip strength was 26.24 ± 3.17 kg, ASMI was 6.16 ± 0.27 kg/m^2^. Accordingly, 17 males and 20 females were diagnosed with sarcopenia.

Table 1 shows the basic characteristics of patients stratified in the DII quartile. Compared to the patients in Quartile 1, patients in the highest quartile (Quartile 4) who consumed a more pro-inflammatory diet may have lower muscle mass and strength, as well as higher CRP scores and a higher prevalence of sarcopenia and malnutrition. Other factors, such as age, sex, smoking, drinking status, disease location and disease behavior, and biomarkers, such as hemoglobin and albumin, revealed no significant differences.

### 3.2. Dietary Intakes of CD Patients across Quartiles of the DII

Table 2 shows the intake of each food parameter in DII calculation across DII quartiles. We observed that CD patients in the first quartile had a high consumption of anti-inflammatory nutrients, such as PUFA, β-carotene, fiber, isoflavones, and other vitamins and minerals, compared to participants in the fourth quartile (*p* < 0.05). Additionally, we found that CD patients in the first quartile (more anti-inflammatory diet) had a higher intake of partial enteral nutrition (PEN) than the fourth quartile (*p* < 0.001).

### 3.3. DII was Associated with Sarcopenia in CD Patients

The overall prevalence of sarcopenia was 26.4% (*n* = 37) with rates of 20%, 20%, 8.57%, and 51.74% across DII quartiles, respectively. In Model 2 and Model 3 (Table 3), compared with the lowest quartiles of DII, the highest quartiles of DII were found to have a greater risk of sarcopenia in CD patients (OR_Quartile4vs1_: 7.22, 95% CI: 1.95, 26.80, *p*_trend_ = 0.009; OR_Quartile4vs1_: 9.59, 95% CI: 1.69, 54.42, *p*_trend_ = 0.031, respectively). In addition, we found that a more pro-inflammatory diet (Quartile 4) had lower ASMI in Model 2, compared with the lowest quartiles of DII (OR_Quartile4vs1_: 5.48, 95% CI: 1.51, 19.87, *p*_trend_ = 0.018). Still, no statistical associations were observed between DII and handgrip strength in the unadjusted and multivariable-adjusted models (model 1, model 2 and model 3). 

## 4. Discussion

“What else can I eat when I have this disease?” This is a common question among CD patients in China. Even though many IBD-related dietary guidelines exist, no specific diet has been established as suitable for IBD patients. Our previous studies have shown that patients with CD often have low energy intake, low protein intake, and were frequently found with malnutrition, disability, and muscle deterioration [9]. In addition, inflammation contributes to the pathogenesis of inflammatory bowel disease and sarcopenia. Therefore, it is crucial to investigate the correlation between inflammation, diet, and sarcopenia in CD patients for disease management and treatment.

Our results revealed that CD patients with a more pro-inflammatory diet (higher DII) had higher CRP. This result further confirmed that DII could reflect inflammatory conditions in the body. Vagianos et al. demonstrated that each unit increase in Empirical Dietary Inflammatory Index (EDII) was associated with an almost 7 unit increase in IBD Symptom Inventory score. Moreover, the EDII could be used to identify the inflammatory potential of diet, which can contribute to inflammation and/or disease symptoms in IBD patients [31]. Diet is a critical environmental factor, with an important role in the homeostasis of the gut microenvironment, which it exerts by regulating gut hormone release of gut hormones, and influencing the gut’s microbial composition and functioning, the gut barrier, host immunity, and the gut physiology [32]. Hence, the underlying mechanism of an inflammatory diet in IBD may be that particular diet components have a direct or indirect role in the development of IBD through 3 physiological processes: the intestinal microbiota, the immune system, and the intestinal barrier [8]. Surprisingly, we found that CD patients with higher DII had lower energy, protein, carbohydrate, and fat intake. This result illustrated that low energy intake was the most serious problem encountered by CD patients. Additionally, de Vries et al. reported that 76.5% IBD patients avoided certain foods to reduce disease symptoms [5]. Chronic reduced food intake inevitably leads to malnutrition, which is more detrimental to the remission of inflammation. Furthermore, we found that the higher DII was associated with, the lower intake of PEN. Enteral nutrition formulas generally contain anti-inflammatory components, such as soy/whey protein, unsaturated fatty acids, rich in vitamins and minerals. Levine et al. reported that a novel Crohn’s disease exclusion diet, coupled with PEN, was better tolerated than exclusive enteral nutrition, resulting in superior sustained remission and reduction in inflammation by week 12. They also found this dietary therapy produced changes in the fecal microbiome associated with remission, decreased in *Haemophilus*, *Veillonella*, *Anaerostipes*, and *Prevotella*, whereas *Roseburia* (an important butyrate producer) and *Oscillibacter* increased [33]. Additionally, Sazuka et al. showed that concomitant use of EN ≥ 600 kcal/day in CD patients might produce a sustained response to infliximab by an 8-year cohort study [34]. We found the average intake of CD patients with the lowest quartile (a more anti-inflammatory diet) was 555.42 ± 397.02 kcal/d, which is consistent with the results of Sazuka [34]. Some studies reported that pro-inflammatory dietary was associated with higher disease activity in IBD patients; however, this view is still controversial [35,36]. Again, this result was not observed in our study.

A recent meta-analysis revealed that 52% of patients with CD had sarcopenia [37]. In order to explore the association between DII and sarcopenia in CD patients more efficiently, we performed a multivariable model that was adjusted for potential factors, such as malnutrition, disease activity, disease location, and behavior etc. In the present study, after adjusting for the multivariable covariate, CD patients in the highest quartile of the DII with a more pro-inflammatory diet had a higher risk of sarcopenia. Besides, we found that the highest quartiles of DII score were associated with a 577% increase in odds of sarcopenia in CD patients after adjusting for the multivariable covariate, even though the *p*-trend showed no statistical significance. Yet, our results showed that DII was not correlated with muscle function in either the unadjusted or adjusted models. Therefore, DII in CD patients is associated with sarcopenia, which is mostly dependent on muscle mass. 

Chronic inflammation is believed to be a key driver in sarcopenia in IBD [11]. Moreover, IBD is usually accompanied by an increase in pro-inflammatory cytokines [38]. Furthermore, inflammatory cytokines have a direct catabolic effect on protein metabolism, reducing muscle protein synthesis and anabolic drive [11]. Other studies showed that gut microbiota was involved in the pathogenesis of sarcopenia in IBD patients. These patients suffered from gut microbiota dysbiosis. During this process, the gut microbiota shifts from protective to pro-inflammatory effects, potentially altering immune responses and host metabolism and promoting a low-grade inflammatory state, thereby up-regulating several molecular pathways associated with sarcopenia and leading to musculoskeletal impairment [10,39]. In addition, we found that CD patients suffered from inadequate energy and protein intake. Our results revealed that CD patients with the highest quartile DII (a more pro-inflammatory diet) had the highest level of CRP, as well as the lowest energy and protein intake. The average protein intake with the highest quartile DII was 43.83 ± 4.74 g/d, which is insufficient according to recent recommendations (at least 1 g/kg/day) for CD patients [40]. This also showed that the most serious problem for CD patients following a more pro-inflammatory diet was inadequate energy and protein intake, which was even more disadvantageous to the remission of inflammation and could exacerbate the loss of muscle mass. As is well known, a prolonged lack of intake or uptake of nutrition can lead to the development of malnutrition. Besides, malnutrition is a key driver for muscle loss and the consequential loss of function leading to sarcopenia. Therefore, adequate energy and protein supplementation are a very important therapeutic measure for patients with CD, which may decrease the inflammation status as well as the risk of sarcopenia.

In China, IBD specialists are more concerned about clinical remission with medication, and even though many tertiary public hospitals have established IBD-MDT for the combined management of IBD patients, nutritional therapy is still under-appreciated. More surprisingly, the dietary recommendations given to patients by IBD specialists are heterogeneous, which increases the confusion of IBD patients, resulting in greater uncertainty about their dietary choices [41]. The guidance by a professional dietitian and the provision of education around alternate sources of key nutrients or supplemental nutrition where appropriate could mitigate the risk of inadequate nutrient intake due to dietary modification [42]. In contrast, the dietitian’s role is not valued, and many IBD specialists and even patients consider them to be redundant in China. It is irrefutable that CD patients in China would benefit from nutrition-related education to prevent malnutrition and sarcopenia due to inadequate intake.

This study has several strengths. To the best of our knowledge, this is the first study that investigated the DII in relation to sarcopenia in Chinese CD patients. Moreover, we used two domains to measure sarcopenia, including muscle mass and muscle function, representing different aspects of sarcopenia. However, there were also some limitations in our study. Firstly, dietary intake was assessed by FFQ, which has limitations in terms of the potential for recall food groups. Secondly, this was a cross-sectional study with small sample size. Moreover, the cohort size was determined only by the number of consecutive outpatients during the sample collection period. We also set relatively strict inclusion and exclusion criteria. Therefore, selection bias could not be excluded and, considering the small sample size, our observations cannot be generalized to all Chinese CD patients.

## 5. Conclusions

A pro-inflammatory diet was associated with a higher risk of sarcopenia. CD patients should have a proper intake of energy and protein. When this intake is inadequate, supplementation with enteral nutrition formulas should be considered, as it may have anti-inflammatory benefits. At the same time, it is important to strengthen the role of dietitians in CD treatment, as they can assist CD patients in maintaining and facilitating proper dietary behaviors.

## Figures and Tables

**Table 1:** Basic characteristics of patients stratified in the DII quartile.

Variable	DII				*p*-Value
	Quartile 1 (*n* = 35)	Quartile 2 (*n* = 35)	Quartile 3 (*n* = 35)	Quartile 4 (*n* = 35)	
	(−3.24, −1.05)	(−1.05, 1.13)	(1.13, 2.67)	(2.67, 4.89)	
Age (years)	32.37 ± 2.75	32.63 ± 4.05	31.43 ± 3.28	33.91 ± 3.93	0.792
BMI (kg/m^2^)	20.29 ± 1.10	21.13 ± 1.04	21.61 ± 1.11	19.76 ± 1.12	0.071
Handgrip strength (kg)	31.43 ± 3.13	30.78 ± 2.94	37.44 ± 3.20	30.91 ± 2.96	0.005 *
Disease duration (years)	4.80 ± 15.4	5.17 ± 1.39	4.49 ± 1.56	7.83 ± 2.43	0.030 *
C-Reactive Protein(mg/L)	3.43 ± 7.68	8.23 ± 15.25	9.53 ± 18.79	15.55 ± 25.36	0.048 *
Hemoglobin(mg/L)	133.83 ± 6.64	130.26 ± 9.18	133.46 ± 7.95	121.91 ± 6.58	0.096
Albumin (mg/L)	42.92 ± 1.54	41.80 ± 2.25	41.15 ± 1.83	41.36 ± 1.95	0.530
ASMI (kg/m^2^)	7.52 ± 0.39	7.33 ± 0.30	7.50 ± 0.41	6.84 ± 0.38	0.034 *
PG-SGA	2.66 ± 0.84	4.32 ± 1.04	3.46 ± 0.90	5.86 ± 1.80	0.002 *
DII	−2.09 ± 0.23	0.06 ± 0.21	1.88 ± 0.16	3.39 ± 0.18	<0.001 *
Male (%)	82.86%	68.57%	71.43%	65.71%	0.406
Smoking status					
Current smoker (%)	2.86%	2.86%	2.86%	8.57%	0.710
Former smoker (%)	8.57%	8.57%	8.57%	8.57%	
Nonsmoker (%)	85.71%	88.57%	85.71%	82.86%	
Drinker (%)	2.86%	0	2.86%	2.86%	0.801
Montreal Classification-Location (%)					
L1	31.14%	51.43%	51.43%	31.43%	0.294
L2	17.14%	22.86%	14.29%	34.28%	
L3	42.86%	25.71%	34.29%	34.28%	
L4	2.86%	0	0	0	
Montreal Classification-Behavior (%)					
B1	60.00%	74.29%	62.86%	68.57%	0.905
B2	34.29%	22.86%	31.43%	28.57%	
B3	5.71%	2.86%	5.71%	2.86%	
Sarcopenia %	20.00%	20.00%	8.57%	57.14%	<0.001 *
Malnutrition %	22.86%	51.43%	42.86%	62.86%	0.006 *
CDAI score	82.30 ± 62.26	57.66 ± 73.60	67.54 ± 76.99	95.82 ± 67.49	0.394
Active phase (%)	17.14%	17.14%	11.42%	14.28%	0.893

* *p* < 0.05, using χ^2^ test for variables of proportion and one-way ANOVA for continuous variables. ASMI, appendicular skeletal muscle mass index. PG-SGA, Patient-Generated Subjective Global Assessment. Montreal Classification-Location, L1: terminal ileum, L2: colon, L3: ileocolon, and L4: involving the upper gastrointestinal tract. Montreal Classification-Behavior, B1: non-stricturing and non-penetrating, B2: stricturing, and B3: penetrating. CDAI, Crohn’s disease activity index, Active phase defined as CDAI ≥ 150.

**Table 2:** Dietary intakes of CD patients across quartiles of the DII.

Food Components (Mean ± SD)	Quartile 1 (*n* = 35) (−3.24, −1.05)	Quartile 2 (*n* = 35) (−1.05, 1.13)	Quartile 3 (*n* = 35) (1.13, 2.67)	Quartile 4 (*n* = 35) (2.67, 4.89)	*p*-Value
PEN (kcal)	555.42 ± 397.02	234.77 ± 191.24	43.31 ± 84.27	18.15 ± 55.25	<0.001 *
Alcohol (g)	0.31 ± 0.64	0 ± 0	0.12 ± 0.25	0.06 ± 0.12	0.591
Vitamin B12 (μg)	8.39 ± 2.19	5.59 ± 1.45	4.19 ± 1.36	6.32 ± 2.42	0.018 *
Vitamin B6 (mg)	2.86 ± 0.24	2.14 ± 0.30	1.52 ± 0.14	1.15 ± 0.15	<0.001 *
β-Carotene (μg)	5156.52 ± 1962.81	3375.32 ± 1459.11	3233.56 ± 1062.02	1516.66 ± 429.70	0.002 *
Carbohydrate (g)	278.88 ± 28.01	244.26 ± 27.44	203.47 ± 19.27	200.43 ± 27.08	<0.001 *
Cholesterol (mg)	552.57 ± 111.37	547.23 ± 86.69	451.38 ± 72.76	369.83 ± 58.37	0.006 *
Energy (kcal)	1953.05 ± 142.23	1775.61 ± 184.59	1421.34 ± 113.24	1192.53 ± 327.51	<0.001 *
Total fat (g)	60.51 ± 6.38	60.60 ± 10.48	44.77 ± 6.97	29.95 ± 5.02	<0.001 *
Saturated fat (g)	16.45 ± 6.03	20.02 ± 17.41	13.90 ± 7.37	9.32 ± 4.93	<0.001 *
Fiber (g)	6.53 ± 1.45	5.20 ± 0.76	5.70 ± 0.79	4.88 ± 0.69	0.085
Folic acid (μg)	541.02 ± 45.25	341.12 ± 22.55	235.04 ± 17.89	184.74 ± 17.41	<0.001 *
Fe (mg)	23.82 ± 3.61	18.53 ± 3.61	14.11 ± 2.73	11.80 ± 1.90	<0.001 *
Mg (mg)	346.57 ± 22.26	260.50 ± 24.63	207.68 ± 13.55	164.59 ± 16.37	<0.001 *
MUFA (g)	18.21 ± 2.32	20.41 ± 4.07	15.97 ± 2.89	9.95 ± 2.01	<0.001 *
Niacin (mg)	26.56 ± 2.14	19.87 ± 1.94	14.74 ± 1.47	10.77 ± 1.26	<0.001 *
n-3 Fatty acids (g)	0.99 ± 0.18	1.13 ± 0.44	0.67 ± 0.23	0.49 ± 0.16	0.004 *
n-6 Fatty acids (g)	16.36 ± 2.30	12.60 ± 4.01	6.27 ± 0.74	5.33 ± 2.78	<0.001 *
Protein (g)	79.26 ± 5.06	68.79 ± 6.86	55.84 ± 4.32	43.83 ± 4.74	<0.001 *
PUFA (g)	10.19 ± 1.09	9.14 ± 1.14	6.60 ± 0.84	4.56 ± 0.93	<0.001 *
Riboflavin (mg)	1.82 ± 0.21	1.11 ± 0.09	0.71 ± 0.08	0.56 ± 0.06	<0.001 *
Saturated fat (g)	16.45 ± 2.07	20.02 ± 5.98	13.90 ± 2.53	9.32 ± 1.70	<0.001 *
Se (μg)	65.59 ± 5.59	52.35 ± 4.78	37.54 ± 3.34	30.42 ± 3.09	<0.001 *
Thiamin (mg)	1.75 ± 0.17	1.23 ± 0.12	0.85 ± 0.10	0.69 ± 0.08	<0.001 *
Vitamin A (RE)	2149.20 ± 338.99	1082.67 ± 125.33	558.26 ± 111.47	381.02 ± 81.47	<0.001 *
Vitamin C (mg)	164.95 ± 16.42	86.94 ± 7.25	63.23 ± 11.99	44.96 ± 8.37	<0.001 *
Vitamin D (μg)	9.11 ± 1.45	5.12 ± 0.73	2.39 ± 0.49	2.21 ± 0.47	<0.001 *
Vitamin E (mg)	22.24 ± 2.61	13.49 ± 1.09	8.74 ± 1.10	6.50 ± 1.08	<0.001 *
Zn (mg)	16.64 ± 1.09	13.11 ± 1.06	9.78 ± 0.69	7.27 ± 0.75	<0.001 *
Isoflavones (mg)	6.36 ± 1.82	5.31 ± 1.66	4.17 ± 1.95	3.11 ± 2.00	0.074

DII, Dietary Inflammatory Index. PEN, partial enteral nutrition. PUFA, polyunsaturated fatty acid. MUFA, Monounsaturated fatty acids. * *p* < 0.05, one-way ANOVA for continuous variables.

**Table 3:** Multivariable adjusted models (95% confidence intervals) for the risk of sarcopenia.

Model	DII, OR (95% Cl)				*p*-Trend
	Quartile 1	Quartile 2	Quartile 3	Quartile 4	
Sarcopenia					
Model 1	1	1.00 (0.31, 3.23)	0.38 (0.09, 1.59)	5.33 (1.84, 15.47)	0.012
Model 2	1	1.24 (0.30, 5.06)	0.45 (0.08, 2.45)	7.22 (1.95, 26.80)	0.009
Model 3	1	1.09 (0.23, 5.04)	0.52 (0.08, 3.41)	9.59 (1.69, 54.42)	0.031
Muscle Mass (ASMI ^a^)					
Model 1	1	0.63 (0.21, 1.89)	0.42 (0.13, 1.38)	4.23 (1.55, 11.55)	0.022
Model 2	1	0.69 (0.18, 2.73)	0.48 (0.11, 2.23)	5.48 (1.51, 19.87)	0.018
Model 3	1	0.63 (0.15, 2.72)	0.51 (0.09, 2.79)	5.77 (1.06, 31.35)	0.106
Muscle Function (Handgrip strength ^b^)					
Model 1	1	0.53 (0.20, 1.44)	0.33 (0.12, 0.97)	2.00 (0.77, 5.18)	0.432
Model 2	1	0.53 (0.18, 1.55)	0.33 (0.10, 1.06)	1.87 (0.67, 5.21)	0.481
Model 3	1	0.48 (0.15, 1.58)	0.23 (0.06, 0.89)	1.26 (0.33, 4.83)	0.804

Odds ratios and 95% confidence interval were estimated with logistic regression models with different levels of adjustment. Model 1 was unadjusted for any covariate. Model 2 was adjusted for sex (male/female), age (continuous) and Body mass index (BMI < 18.5 kg/m^2^, 18.5 ≤ BMI ≤ 24 kg/m^2^, BMI > 24 kg/m^2^), smoking status (current/former/never), alcohol consumption (no/moderate/regular). Model 3 was additionally adjusted for nutritional status (malnutrition: PG-SGA ≥ 4, normal nutritional status: PG-SGA < 4), disease activity (active phase: CDAI ≥ 150, remission phase: CDAI < 150), total energy intake, disease duration, Montreal Classification-Location (L1: terminal ileum, L2: colon, L3: ileocolon, and L4: involving the upper gastrointestinal tract) and Montreal Classification-Behavior (B1: non-stricturing and non-penetrating, B2: stricturing, and B3: penetrating). The trend is calculated with the quartile number as a continuous variable. ^a^ ASMI (appendicular skeletal muscle mass index, ASMI < 7.0 kg/m^2^ for male, ASMI < 5.7 kg/m^2^ for female, via BIA) ^b^ Handgrip strength < 28 kg for male, handgrip strength < 18 kg for female.

## Data Availability

Not applicable.
